# Prognostic Factor Analysis of Intraocular Pressure with Neovascular Glaucoma

**DOI:** 10.1155/2016/1205895

**Published:** 2016-08-14

**Authors:** Satoko Nakano, Takako Nakamuro, Katsuhiko Yokoyama, Kunihiro Kiyosaki, Toshiaki Kubota

**Affiliations:** Department of Ophthalmology, Oita University Faculty of Medicine, 1-1 Idaigaoka, Hasama-machi, Yufu-City, Oita 879-5593, Japan

## Abstract

*Purpose*. To perform multivariate analysis for identifying independent predictors of elevated intraocular pressure (IOP) with neovascular glaucoma (NVG), including antivascular endothelial growth factor (VEGF) intravitreal injections.* Methods*. We retrospectively reviewed 142 NVG patients (181 eyes) with ischemic retinal diseases [proliferative diabetic retinopathy (PDR) in 134 eyes, retinal vein occlusion (RVO) in 29, and ocular ischemic syndrome in 18]. We analyzed age, gender, initial/final LogMAR VA, initial/final IOP, extent of iris and/or angle neovascularization, treatments, preexisting complications, concurrent medications, and follow-up duration.* Results*. The mean follow-up duration was 23.8 ± 18.8 months. At the final follow-up, 125 (72.3%) eyes had IOP ≤ 21 mmHg. NVG patients with RVO had a higher degree of angle closure and higher IOP. NVG with PDR had better IOP and LogMAR VA. Angle closure had the greatest impact on final IOP. Greater than 90% of patients treated with trabeculectomy with mitomycin C (LEC) had persistent declines in IOP (≤21 mmHg). Stand-alone and combination anti-VEGF therapies were not associated with improved long-term prognosis of IOP.* Conclusions*. Angle closure was found to have the greatest effect on NVG-IOP prognosis. When target IOP values are not obtained after adequate PRP with or without anti-VEGF, early LEC may improve the prognosis of IOP.

## 1. Introduction

Neovascular glaucoma (NVG) is a severe consequence of a number of ocular and systemic conditions, such as proliferative diabetic retinopathy (PDR), retinal vein occlusion (RVO), ocular ischemic syndrome (OIS), tumors, trauma, or uveitis [1]. It is indisputable that increased awareness of risk factors and the early detection of retinal ischemia can minimize poor prognosis of NVG; however, many NVG patients suffer loss of vision as a result of irreversible high intraocular pressure (IOP) despite the use of preexisting treatments, such as panretinal laser photocoagulation (PRP), pars plana vitrectomy (PPV), and trabeculectomy with mitomycin C (LEC) [2]. The main causative factor of NVG is retinal hypoxia that initiates the subsequent release of angiogenesis factors, and NVG is characterized by changes in the appearance of the iris, angle closure glaucoma, and formation of new vessels [1]. Gartner and Henkind showed that the main mechanism of intraocular pressure elevation was angle closure, with peripheral anterior synechia [3]. However, NVG occurs when new fibrovascular tissue proliferates onto the iris and chamber angle structures, including the trabecular meshwork, typically in response to ischemia of various etiologies [2, 4, 5]. Previous studies have demonstrated increased production of several proangiogenic factors, including vascular endothelial growth factor (VEGF) [6]. In addition to the ischemic retina, nonpigmented ciliary epithelium and iris contribute to VEGF synthesis in NVG patients [7]. The created fibrovascular neovascularizational membrane promoted by these proangiogenic factors inhibits the aqueous flow and leads to an increase in IOP [4]. Recent studies on intravitreal injection of anti-VEGF agents as stand-alone or as combination treatment with other NVG treatments have reported excellent results [8–12]; however, responses to treatment with a single injection are considered temporary [13, 14]. Anti-VEGF treatment was introduced in our university hospital in September 2006. We compared the cause-specific prognosis of NVG and efficacy between treatments. Further, we performed multivariate analysis to identify NVG prognostic factors for IOP.

## 2. Subjects and Methods

### 2.1. Subjects

We performed a retrospective study of 142 NVG patients (181 eyes) with ocular ischemic diseases who had visited the Oita University Hospital between September 2006 and May 2014 (follow-up duration: mean ± STD, 23.8 ± 18.8 months; range: 4.0–81.1 months). All procedures were performed in accordance with the Declaration of Helsinki.

### 2.2. Diagnostic Criterion for NVG

All patients underwent full ocular examinations, including undilated gonioscopy and pupil examinations [2]. NVG was staged according to the degree of neovascularization, angle closure, and intraocular pressure (IOP). Anterior segment fluorescein angiography (FA) and indocyanine green angiography (IA) for iris and angle neovascularization were used to confirm the presence of newly formed vessels. NVG staging criteria in this study were as follows: rubeosis group, angle and/or iris neovascularization only without peripheral anterior synechia (PAS) and normal IOP; open-angle NVG group, open angle and high IOP (>21 mmHg) due to neovascularization; and angle closure NVG group, closed angle and high IOP (>21 mmHg) with PAS.

### 2.3. Treatment Plan of NVG

**Figure 1:**
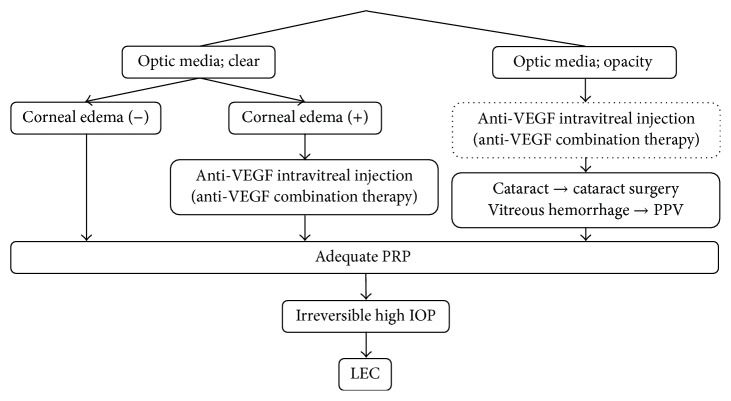
Treatment plan of NVG in Oita University Hospital. A variety of treatments were administered in the present study depending on NVG patient status, including panretinal laser photocoagulation (PRP), anti-VEGF intravitreal injection (stand-alone or in combination with other treatments), cataract surgery, pars plana vitrectomy (PPV), and trabeculectomy with 0.02% mitomycin C (LEC). PRP, panretinal laser photocoagulation; PPV, pars plana vitrectomy, LEC, trabeculectomy with mitomycin C; VEGF, vascular endothelial growth factor.

NVG patients with clear optic media without corneal edema were treated with adequate PRP. Adequate PRP was defined as more than 3000 laser burns sufficient for disappearance of nonperfusion area (NPA). For patients with clear optic media and corneal edema, we performed adequate PRP after anti-VEGF (bevacizumab [11, 14–18] or ranibizumab [8, 9, 19]) intravitreal injections (0.5 mg/0.05 mL). For patients with severe ischemic retinopathy, anti-VEGF was also injected in anticipation of NPA reduction after adequate PRP. Bevacizumab was used before the approval of ranibizumab. Patients administered bevacizumab were informed regarding the off-label use of these drugs in the majority of cases at the time of injection and the approval of their use by the Institutional Review Board of Oita University. Patients with optic media opacity were first treated with cataract surgery or PPV before adequate PRP. LEC was performed after adequate PRP in patients with high IOP. Anti-VEGF intravitreal injections were occasionally combined with the treatments listed above. Combination therapy was defined as single anti-VEGF intravitreal injection within 2 weeks of other treatments. A proportion of patients were treated with stand-alone anti-VEGF intravitreal injections according to patient preference when IOP elevation or neovascularization exacerbation was observed (Figure 1). Concurrent administration of medications, such as systemic acetazolamide and combination eye drops, with all other treatments was performed as required.

### 2.4. Observation Items

Observation items were age, gender, initial LogMAR VA (visual acuity), initial IOP, the extent of newly formed vessels in iris and/or angle, previous treatments, preexisting complications, treatments, final LogMAR VA, final IOP, concurrent medications, and follow-up duration. IOP was measured using a Goldmann applanation tonometer in the presence of concurrent medication.

### 2.5. Primary and Secondary Outcome Measurements

The primary outcome of the present study was final IOP. Secondary outcomes were LogMAR VA and number of concurrent medications. NVG cause-specific final IOP, LogMAR VA, and patient backgrounds were evaluated in the present study. IOP and the number of concurrent medications at 4 months after each treatment, including stand-alone anti-VEGF, additional PRP, PPV, and LEC, were also analyzed. Further, long-term IOP prognosis associated with anti-VEGF and intravitreal injection combination therapy within 2 weeks of other treatments was also evaluated. Finally, we conducted multivariate statistical analyses to identify IOP prognostic factors of NVG.

### 2.6. Statistical Analyses

Comparisons between cause-specific NVG patient groups and treatment groups were assessed by one-way repeated measures analysis of variance (ANOVA) and the Steel-Dwass test. IOP after each treatment was analyzed using the Kaplan-Meier test (end point; IOP > 21 mmHg). After-treatment IOP with or without anti-VEGF combination therapy was analyzed using the paired-*t* test for pre-treatment IOP. Long-term prognosis was compared between the presence and absence of anti-VEGF combination therapy using the Mann-Whitney *U* test and Kaplan-Meier methods (end point; IOP > 21 mmHg). Log-rank test and Cox proportional-hazards models were created to identify prognostic factors of NVG using final IOP > 21 mmHg as the study end point. Causative disease (PDR, RVO, and OIS), angle closure, previous treatments (PRP, PPV), preexisting complications (hyphema, vitreous hemorrhage), and treatments (additional PRP, PPV, LEC, and anti-VEGF agents) were included as covariates. IOP, age, LogMAR VA, and follow-up durations were presented as means ± SD. *P* < 0.05 was considered statistically significant. All statistical analyses were performed using SPSS Statistics 23 (IBM, New York) and JMP11 (SAS Institute Inc., Cary, NC).

## 3. Results

### 3.1. Patients

We enrolled 142 patients (181 eyes) with NVG due to ocular ischemic disease. Underlying retinal diseases included PDR in 134 eyes, RVO in 29, and OIS in 18. The mean follow-up duration was 23.8 ± 18.8 months (range, 4.0–81.1 months). All patients at initial visits had nonperfusion areas (NPA) on FA. At the final follow-up visit, all patients were confirmed to have no evidence of NPA on FA following treatment, with IOP ≤ 21 mmHg observed in 125 (72.3%) eyes. No serious adverse events were observed with any treatments in the present study.

### 3.2. Causes and Prognosis

**Figure 2:**
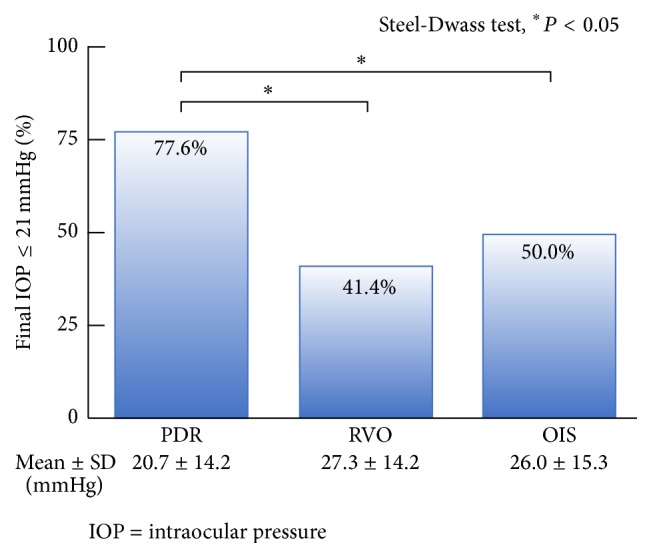
Final IOP values according to NVG causation. NVG patients with PDR had better IOP values than others.

**Figure 3:**
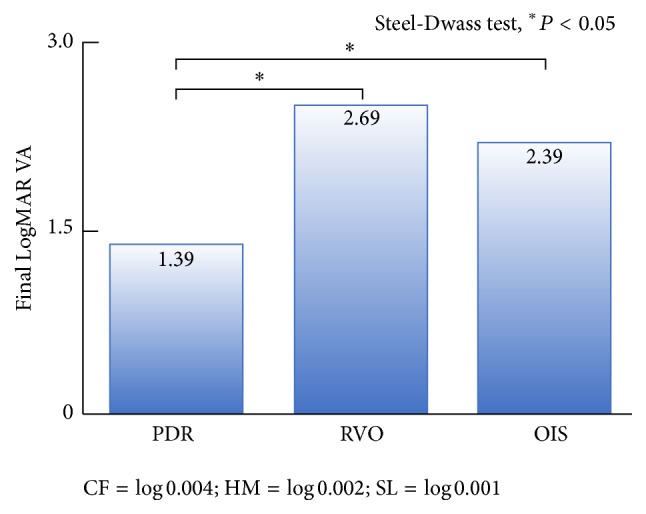
Final LogMAR VA values according to NVG causation. The majority of NVG patients had substantially lower final LogMAR VA; however, NVG patients with PDR had comparatively better final LogMAR VA than others.

The mean follow-up duration was 26.2 ± 22.1 months (range, 4.0–81.1 months) in PDR patients, 17.6 ± 18.8 months (range, 4.0–70.1 months) in RVO patients, and 16.5 ± 13.0 months (range, 4.0–40.0 months) in OIS patients. NVG patients with PDR were younger and had a higher PRP ratio (81/134, 60.4%) than the RVO (8/29, 27.6%) and OIS groups (5/18, 27.8%). NVG patients with RVO had a greater closed-angle ratio (angle closure NVG group, 17/29, 58.6%) and higher IOP (42.4 ± 13.8 mmHg) than the PDR (45/134, 33.6%, 36.4 ± 13.8 mmHg) and OIS groups (7/18, 38.9%, 35.0 ± 11.9 mmHg). Patients in the NVG with OIS group had a higher incidence of hyphema (6/18, 33.3%) than other groups (Table 1). In the analysis of NVG cause-specific final IOP, NVG patients with PDR had lower IOP (20.7 ± 14.2 mmHg) than the NVG with RVO (27.3 ± 14.2 mmHg) and OIS (26.0 ± 15.3 mmHg) groups (Figure 2). The majority of NVG patients had substantially lower final LogMAR VA values (1.71 ± 1.55); however, the NVG with PDR group had better LogMAR VA values (1.39 ± 1.45) compared with the those of the RVO (2.69 ± 1.43) and OIS (2.39 ± 1.68) groups (Figure 3). All vitreous hemorrhages were surgically removed and did not lead to vision loss. Severe vision loss cases (final LogMAR VA, 1.0) were 49.2% (66/134) in PDR, 86.2% (25/29) in RVO, and 66.7% (12/18) in OIS; all of them had optic atrophy. The causes for the modest vision loss (final LogMAR VA, 0.3 to 1.0) were macular edema or corneal edema.

### 3.3. Treatments and Prognosis

**Figure 4:**
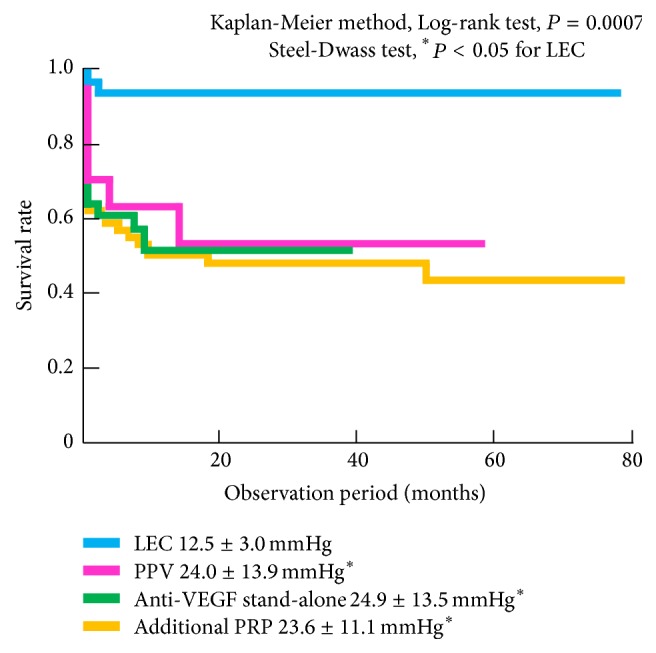
IOP progression according to by NVG treatment. LEC had the strongest continuous hypotensive effect, resulting in persistent declines in IOP (≤21 mmHg) in more than 90% of patients. IOP often increased several months after treatment, except following LEC.

**Figure 5:**
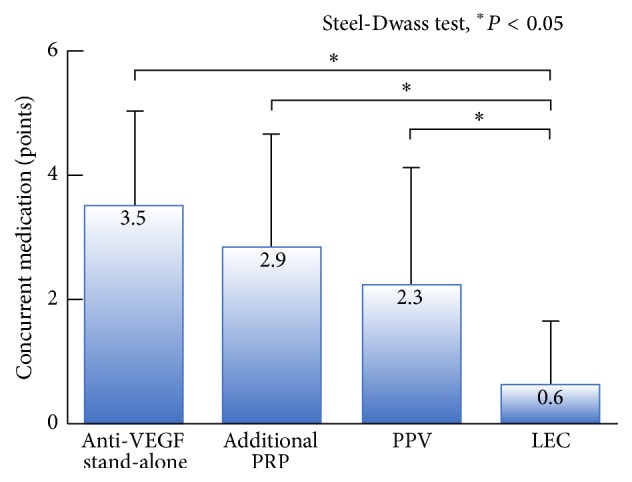
Number of concurrent medications according to NVG treatment received. LEC was associated with the lowest number of concurrent medications compared with other treatments. Concurrent medications were weighted as follows: systemic acetazolamide, 2 points; eye drops, 1 point; and mixed eye drops, 2 points.

The mean follow-up durations pre- and post-treatment are shown in Table 2. PRP was administered to all patients who received stand-alone anti-VEGF therapy. Approximately half (7/17, 41.2%) of these patients had previously received anti-VEGF injections (mean number of injections, 11.1 ± 10.4, range, 3–31; mean duration, 2.0 ± 1.1 months, range 0.7–3.9 months). Forty-nine patients (55.1%) in the additional PRP group, 15 (53.6%) in the PPV group, and 3 (9.4%) in the LEC group received anti-VEGF combination therapy. We performed LEC at a median time of 7.2 ± 11.5 months after initial visits. Twenty-three patients (71.9%) in the LEC group had angle closure glaucoma. Patients in the LEC group had previously received anti-VEGF therapy (23/32, 71.9%), PPV (7/32, 21.9%), and adequate additional PRP (32/32, 100.0%). Only 1 patient (3.1%) in the LEC group underwent repeat surgery (Table 2). We compared IOP and the number of concurrent medications at 4 months after each treatment. All treatments had a significant effect on IOP. LEC had the strongest hypotensive effect among all the treatments, resulting in persistent declines in IOP in 93.8% (30/32) of patients (mean 24.5 ± 22.6 months; range, 4.3–60.7 months) (Figure 4). IOP progression and bleb survival rate after LEC had comparable Kaplan-Meier curves (data not shown). Stand-alone anti-VEGF therapy, additional PRP, and PPV also resulted in decreased IOP; however, IOP often increased several months after these treatments (Figure 4). LEC was associated with the lowest use of concurrent medications (Figure 5).

### 3.4. Anti-VEGF Combination Therapy and Prognosis

**Figure 6:**
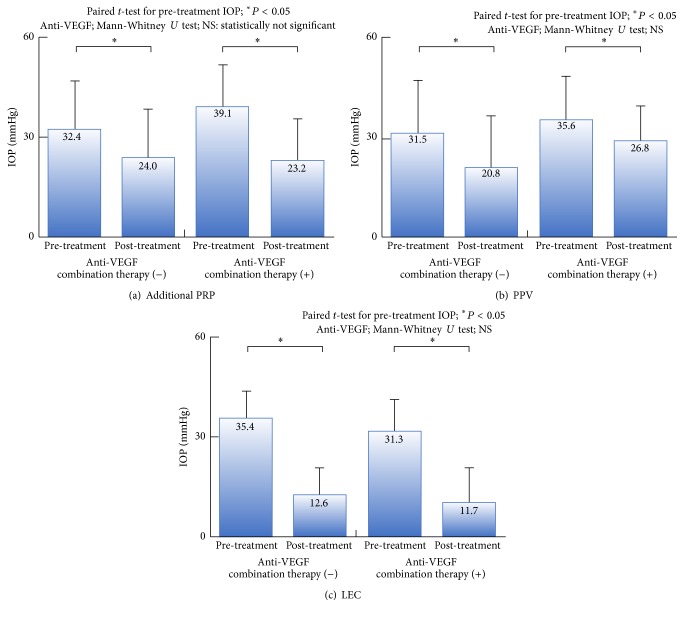
Post-treatment IOP with or without anti-VEGF combination therapy. All treatments with or without anti-VEGF combination therapy had a significant effect on IOP. No significant differences in post-treatment IOP were observed between patients treated with or without anti-VEGF combination therapy.

**Figure 7:**
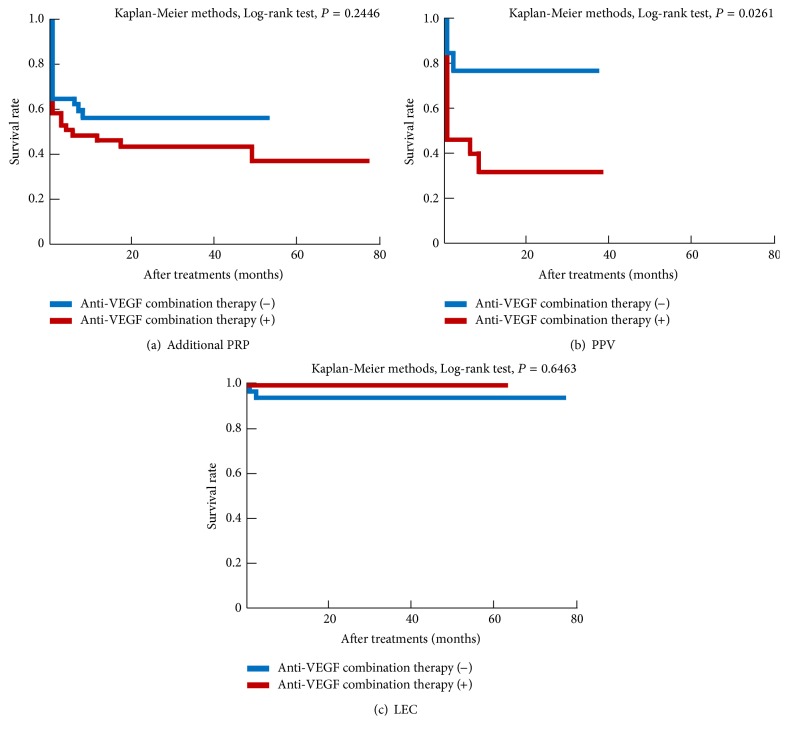
Long-term prognosis of each treatment with or without anti-VEGF combination therapy (univariate analysis). Anti-VEGF combination therapy had no positive impact on long-term prognosis.

All treatments with or without anti-VEGF combination therapy had a significant effect on IOP. No significant differences in the post-treatment IOP were observed between patients treated with or without anti-VEGF combination therapy (Figure 6). When we examined long-term IOP prognosis after additional PRP and LEC using univariate analysis, no differences were observed between patients treated with or without anti-VEGF combination therapy (Figure 7).

### 3.5. Multivariate Statistics and Prognosis

Finally, we evaluated factors influencing final IOP using multivariate statistics. The results are shown in Table 3. Angle closure was found to have the greatest effect on final IOP (hazard ratio 3.059; 95% confidence interval 1.898–4.916), followed by PDR (0.759; 0.391–0.930).

## 4. Discussion

In this study, major cause of irreversible severe visual loss was optic atrophy. NVG with PDR had a better prognosis in terms of IOP and LogMAR VA at the final visit. Many PDR patients could escape optic atrophy because of higher PRP ratio and comparatively restricted ischemic retinal areas at the initial visit than others. Patients with NVG as a result of RVO with broad ischemia had a greater angle closure ratio, higher IOP, and worse VA prognosis.

Regarding treatment-specific prognosis, LEC had the strongest hypotensive effect compared to other treatments, with long-term decreases in IOP maintained in 93.8% of patients. During this study duration, we were unable to evaluate superior anti-VEGF compounds that have since become available, such as aflibercept, and devices, such as the tube surgical treatment option. Our superior LEC-IOP outcomes compared with those of previous studies [20] may be due to the high bleb survival rate as a result of lack of active neovascularization despite angle closure. Adequate PRP resulted in the resolution of NPA on fluorescein angiography in those patients. Full PRP followed by LEC is known to have efficacy in reducing elevated IOP associated with NVG [21]. In addition, LEC contributes to the quality of life of NVG patients by requiring the lowest combination eye drops compared to other treatments. Although other treatments may decrease IOP in the short-term, IOP often increases several months after treatment. Previous studies have reported that NVG often recurs within 1 year of treatment [22]. While additional PRP with anti-VEGF therapy and additional PRP combined with surgery are accepted as important treatments, angle closure NVG group is thought to require LEC. Approximately half of patients in the stand-alone anti-VEGF therapy group required repeated injections in the present study. Anti-VEGF intravitreal injections are reported to have efficacy in inducing the regression of new vessels, although this effect appears to be temporary [15, 22].

Intravitreal injection of anti-VEGF agents in patients with NVG reportedly causes reduced vascular permeability, decreased inflammatory reaction, loss of vascular function, and endothelial cell degeneration [14, 16]. On iris-angle angiography, dye leakage on fluorescein angiography is decreased after intravitreal injection of anti-VEGF agents. Vascular structures in the iris and angle can be observed with indocyanine angiography; however, intravitreal injection of anti-VEGF agents has no effect on these structures despite reports indicating the disappearance of newly formed vessels examined using a slit lamp [17]. Histopathological changes in the trabecular meshwork in NVG following intravitreal injection of anti-VEGF agents revealed that vascular endothelial cells were still present in the trabecular meshwork and fenestrations disappeared [14]. Therefore, repeated stand-alone anti-VEGF injections are necessary to control IOP in NVG eyes with residual retinal ischemia. In the present study, the repeated stand-alone anti-VEGF group consisted of a small number of who refused adequate PRP and LEC due to poor general condition or for psychological reasons.

Anti-VEGF combination therapy was found to have no effect on the prognosis of NVG prognosis in terms of IOP control in the present study. The IOP prognosis of PPV with anti-VEGF combination therapy was worse than that without anti-VEGF combination therapy according to the results of univariate analysis. This finding may be attributable to the use of anti-VEGF combination therapy in severe cases of retinal neovascularization in the present study. A previous study reported IVB increased surgical success rates by decreasing risk of perioperative bleeding [23]; however, other studies have that IVB does not improve long-term prognosis [15, 18]. The efficacy of anti-VEGF combination therapy in improving surgical IOP outcomes remains controversial; however, it remains an accepted therapy for reducing perioperative surgical complications [18].

The present study has certain limitations. We were unable to perform a randomized study due to the retrospective design. We are planning a prospective investigator initiated trial in the future. Furthermore, we cannot try other now available superior anti-VEGF compounds [8] such as aflibercept [19] and devises like the tube surgical option of treatment [24–26] due to disapproval at that time. In addition, as LEC was performed after various treatments, including adequate PRP (100%), anti-VEGF stand-alone therapy (71.9%), and PPV (21.9%), it should be noted that extensive preparation is necessary for the success of LEC. Our follow-up duration after the last LEC treatment (mean 24.5 ± 22.6 months) may have been inadequate for accurate assessment of long-term prognosis.

## 5. Conclusions

We summarize our present results from the retrospective study involving 142 NVG patients. Angle closure was found to have the greatest effect on NVG-IOP prognosis. Therefore, LEC with survival blebs after other adequate treatments, including anti-VEGF treatments, appears to be the effective treatment for NVG. Anti-VEGF combination therapy had no effect on long-term NVG-IOP prognosis but is recommended prior to angle closure. In patients where the target IOP is not obtained following adequate PRP with/without the use of anti-VEGF agents, early LEC may improve the prognosis of NVG-IOP this time, and a high index of suspicion based on patient history and early recognition of high risk eyes are crucial for favorable long-term outcomes. Moreover, NVG treatments are rapidly evolving with time. The randomized prospective study including newest IOP-lowering devises and drugs may be necessary for next prospective study in the future.

## Figures and Tables

**Table 1:** Cause-specific NVG patient backgrounds. NVG patients with proliferative diabetic retinopathy (PDR) were younger and had a higher pre-treatment PRP ratio. NVG patients with retinal vein occlusion (RVO) had a higher incidence of angle closure glaucoma and higher IOP than other groups. Hyphema occurred more frequently in NVG patients with ocular ischemic syndrome (OIS).

Causative ocular ischemic disease	PDR	RVO	OIS
Number of eyes	134	29	18

Age (years)	60.1 ± 11.4	72.2 ± 15.2^*∗*^	71.8 ± 11.3^*∗*^

Both eyes affected (*n*)	38 (39.6%)	0 (0%)	1 (5.9%)

Initial LogMAR VA	1.34 ± 1.06	2.43 ± 1.02	1.98 ± 1.40

Initial IOP (mmHg)	36.4 ± 13.8	42.4 ± 13.8^*∗*^	35.0 ± 11.9

Criteria			
Rubeosis group	17 (12.7%)	0 (0%)	3 (16.7%)
Open-angle NVG group	72 (53.7%)	12 (41.4%)	8 (44.4%)
Angle-closure NVG group	45 (33.6%)	17 (58.6%)^*∗*^	7 (38.9%)

Previous treatment			
PRP	81 (60.4%)	8 (27.6%)^*∗*^	5 (27.8%)^*∗*^
PPV	27 (20.1%)	2 (6.9%)	21 (72.4%)

Preexisting complication			
Hyphema	7 (5.2%)^†^	2 (6.9%)^†^	6 (33.3%)
VH	39 (29.1%)	5 (17.2%)	4 (22.2%)

Follow-up (months)	26.2 ± 22.1 4.0–81.1	17.6 ± 18.8 4.0–70.1	16.5 ± 13.0 4.0–40.0

PDR, proliferative diabetic retinopathy; RVO, retinal vein occlusion; OIS, ocular ischemic syndrome; VA, visual acuity; NVG, neovascular glaucoma; PRP, panretinal laser photocoagulation; PPV, pars plana vitrectomy, VH, vitreous hemorrhage; CF = log⁡0.004; HM = log⁡0.002; SL = log⁡0.001.

Mean ± SD, Steel-Dwass test; ^*∗*^ *P* < 0.05 for PDR, ^†^ *P* < 0.05 for OIS.

**Table 2:** NVG patient backgrounds according to treatment received. The majority of patients in the LEC group had stage 3 NVG and had previously received other treatments, such as adequate PRP, stand-alone anti-VEGF therapy, and PPV. Approximately half (41.2%) of patients that received frequent stand-alone anti-VEGF treatment had previously received repeated anti-VEGF injections.

Treatments	Anti-VEGF stand-alone therapy	Additional PRP	PPV	LEC
Number of treatments	17	89	28	32

Anti-VEGF combination therapy (*n*)	—	49	15	3

Pre-treatment IOP (mmHg)	36.1 ± 12.5	36.1 ± 13.5	33.7 ± 13.9	35.0 ± 8.1

Criteria				
Rubeosis group	1 (5.9%)	9 (10.1%)	4 (14.3%)	1 (3.1%)
Open-angle NVG group	10 (58.8%)	51 (57.3%)	12 (42.9%)	8 (25.0%)
Angle-closure NVG group	6 (35.3%)	12 (32.6%)	12 (42.9%)	23 (71.9%)^*∗*^

Previous treatment				
Anti-VEGF stand-alone therapy	7 (41.2%)^*∗*^	1 (1.1%)	3 (10.7%)^†^	23 (71.9%)^*∗*^
PRP	17 (100.0%)^*∗*^	(44.9%)	(85.7%)	32 (100.0%)^*∗*^
PPV	4 (23.5%)	0 (0%)	0 (0%)	7 (21.9%)
LEC	1 (5.9%)	0 (0%)	0 (0%)	1 (3.1%)

Follow-up (pre-treatment, months)	2.5 ± 2.8^*∗*^ 0.0 to 8.3	0.8 ± 3.0 0.0 to 22.3	3.2 ± 7.9^*∗*^ 1.0 to 33.3	7.2 ± 11.5^*∗*^ 0.0 to 48.8

Follow-up (post-treatment, months)	21.0 ± 19.7 4.1–53.2	25.4 ± 21.6 4.0–81.8	25.0 ± 14.3 5.1–56.0	24.5 ± 22.6 4.3–60.7

PRP, panretinal laser photocoagulation; PPV, pars plana vitrectomy, LEC, trabeculectomy with mitomycin C.

Mean ± SD, Steel-Dwass test ^*∗*^ *P* < 0.05 for PDR, ^†^ *P* < 0.05 for OIS.

**Table 3:** Prognostic factors influencing final IOP in patients with NVG (multivariate statistics). Log-rank test and Cox proportional-hazards models were created to identify prognostic factors of NVG using final IOP > 21 mmHg as the study end-point. Angle-closure was associated with a 3-fold worsening in NVG-IOP prognosis. Patients with NVG with PDR had relatively better prognosis than those with NVG induced by other causes.

Covariates	Hazard ratio	95% confidence interval	Log-rank test (^*∗*^ *P* < 0.05)
Angle-closure NVG group	3.059	1.898–4.916	0.0002^*∗*^
Causative disease, PDR	0.759	0.391–0.930	0.0002^*∗*^
Treatments, LEC	0.412	0.251–0.667	0.0809
Causative disease, RVO	—	—	0.0123^*∗*^
Causative disease, OIS	—	—	0.0384^*∗*^
Previous treatments, PRP	—	—	0.1667
Previous treatments, PPV	—	—	0.2717
Pre-existing complications, hyphema	—	—	0.3930
Pre-existing complications, VH	—	—	0.8108
Treatments, anti-VEGF therapy	—	—	0.3128
Treatments, additional PRP	—	—	0.3642
Treatments, PPV	—	—	0.9287

NVG, neovascular glaucoma; PDR, proliferative diabetic retinopathy; LEC, trabeculectomy with mitomycin C; RVO, retinal vein occlusion; OIS, ocular ischemic syndrome; PRP, panretinal laser photocoagulation; PPV, pars plana vitrectomy; VH, vitreous hemorrhage.
